# MR Prediction of Liver Function and Pathology Using Gd-EOB-DTPA: Effect of Liver Volume Consideration

**DOI:** 10.1155/2015/141853

**Published:** 2015-11-01

**Authors:** Dai Shimamoto, Akihiro Nishie, Yoshiki Asayama, Yasuhiro Ushijima, Yukihisa Takayama, Nobuhiro Fujita, Ken Shirabe, Tomoyuki Hida, Yuichiro Kubo, Hiroshi Honda

**Affiliations:** ^1^Department of Clinical Radiology, Graduate School of Medical Sciences, Kyushu University, 3-1-1 Maidashi, Higashi-ku, Fukuoka 812-8582, Japan; ^2^Department of Surgery and Science, Graduate School of Medical Sciences, Kyushu University, 3-1-1 Maidashi, Higashi-ku, Fukuoka 812-8582, Japan; ^3^Department of Anatomic Pathology, Graduate School of Medical Sciences, Kyushu University, 3-1-1 Maidashi, Higashi-ku, Fukuoka 812-8582, Japan

## Abstract

*Purpose*. To evaluate whether the diagnostic performance of Gd-EOB-DTPA-enhanced MRI in evaluating liver function and pathology is improved by considering liver volume (LV). *Methods*. This retrospective study included 104 patients who underwent Gd-EOB-DTPA-enhanced MRI before liver surgery. For each patient, using the precontrast and hepatobiliary phase images, we calculated the increase rate of the liver-to-spleen signal intensity ratio (LSR), that is, the “ΔLSR,” and the increase rate of the liver-to-muscle signal intensity ratio (LMR), that is, the “ΔLMR.” ΔLSR × LV and ΔLMR × LV were also calculated. The correlation of each MR parameter with liver function data or liver pathology was assessed. The correlation coefficients were compared between ΔLSR (ΔLMR) and ΔLSR (ΔLMR) × LV. *Results*. The correlation coefficient between ΔLSR (ΔLMR) × LV and cholinesterase was significantly higher than that between ΔLSR (ΔLMR) and cholinesterase. The correlation coefficient between ΔLSR (ΔLMR) × LV and the degree of fibrosis or necroinflammatory activity was significantly lower than that between ΔLSR (ΔLMR) and the degree of fibrosis or necroinflammatory activity. *Conclusion*. The inclusion of liver volume may improve Gd-EOB-DTPA-based predictions of liver function, but not in predictions of liver pathology.

## 1. Introduction

Gadolinium ethoxybenzyl diethylenetriamine penta-acetic acid (Gd-EOB-DTPA) is a liver-specific agent, and it is widely used to improve both the detection rate of focal liver lesions and the characterization of liver tumors on magnetic resonance imaging (MRI) [1, 2]. As Gd-EOB-DTPA is taken up specifically by hepatocytes, the measurement of the uptake of Gd-EOB-DTPA in the liver can be used to evaluate liver function [3–5]. A correlation between the uptake of Gd-EOB-DTPA and pathological liver fibrosis has also been reported [6, 7]. That is, the signal intensity itself or the signal intensity change in the hepatobiliary phase decreases as the liver function or fibrosis worsens. In these previous studies, only the degree of Gd-EOB-DTPA uptake on a single slice or several slices was considered as an indicator of liver function or fibrosis. However, the liver volume (LV) is quite different among individuals. We hypothesized that the liver function or fibrosis could be more precisely estimated by using a parameter including the LV, which would represent the whole liver function.

The purpose of the present study was to evaluate whether the diagnostic performance of Gd-EOB-DTPA-enhanced MRI in evaluating liver function or fibrosis is improved by considering the LV.

## 2. Methods

### 2.1. Patients

This study was approved by the institutional review board of our hospital. The requirements for informed consent were waived for this retrospective study. Referring to the medical data recorded at our hospital, we enrolled 129 consecutive patients who underwent Gd-EOB-DTPA-enhanced MRI and hepatic resection for a liver tumor or liver transplantation between June 2010 and May 2013. Of them, twelve, eight, and five patients were excluded due to a history of splenectomy, a history of right or left lobectomy, and poor image quality derived from respiratory artifacts, respectively. Finally, 104 patients were enrolled in this study. The 104 patients included 69 men and 35 women (age range, 32–86 years; mean age, 64.5 years). The hepatitis C virus antibody was present in 45 cases, the hepatitis B surface antigen in 17 cases, alcoholic hepatitis in five cases, nonalcoholic steatohepatitis in five cases, primary biliary cirrhosis in two cases, autoimmune hepatitis in one case, and primary sclerosing cholangitis in one case. The grading of liver dysfunction was preoperatively evaluated based on the Child-Pugh classification, and 86, seven, and 11 patients were categorized into Grades A, B, and C, respectively. The grading of liver function or severity of liver cirrhosis in patients with chronic liver disease was evaluated according to the Child-Pugh classification [8]. The classification is based on the following five factors, graded on a scale from 1 to 3: hepatic encephalopathy, ascites, total bilirubin level, albumin level, and prothrombin time. The liver function or severity of cirrhosis was classed into three groups according to the sum of the scores: Grade A, from 5 to 6; Grade B, from 7 to 9; Grade C, from 10 to 15. The laboratory data were obtained at least within one month before surgery. For each patient, the platelet count (Plt), albumin (Alb), total bilirubin (T-bil), lactate dehydrogenase (LDH), cholinesterase (ChE), Child-Pugh score, and model for end-stage liver disease (MELD) score were recorded. An MR examination was performed at least 3 months before the surgery. No treatment was performed between the MR examination and the surgery for any of the patients.

### 2.2. MR Imaging

MR imaging was performed on a whole-body 3.0 Tesla (T) scanner (Achieva 3.0Tx, Philips Medical Systems, Best, Netherlands). For the Gd-EOB-DTPA-enhanced MRI, axial 3D eTHRIVE (three-dimensional enhanced-T1 high-resolution isotropic volume excitation) was scanned before and 20 min after an intravenous injection of 0.1 mL/kg (total amount: 4 to 8 mL) of Gd-EOB-DTPA (Primovist; Bayer, Osaka, Japan). The detailed imaging parameters were as follows: 32-channel cardiac phased-array coil, TR/TE/FA = 3 ms/1.4 ms/10°, matrix 252 × 200, FOV 37.5 × 29.8 cm, SENSE factor 1.8, slice thickness = 3 mm, gap = −1.5 mm, linear *k*-space ordering, spectral attenuation with inversion recovery, acquired 133 sections, scan time 17.9 s, and breath-holding.

### 2.3. Liver Volume Measurement

For the LV measurement, the total of the MR images in the hepatobiliary phase was prepared for each patient. The LV of each patient was semiautomatically measured using the “liver analysis” function of the volume analyzer SYNAPSE VINCENT (Fuji Film Medical, Tokyo). A part of liver tumor was not considered as LV.

### 2.4. MR Image Analysis

**Figure 1:**
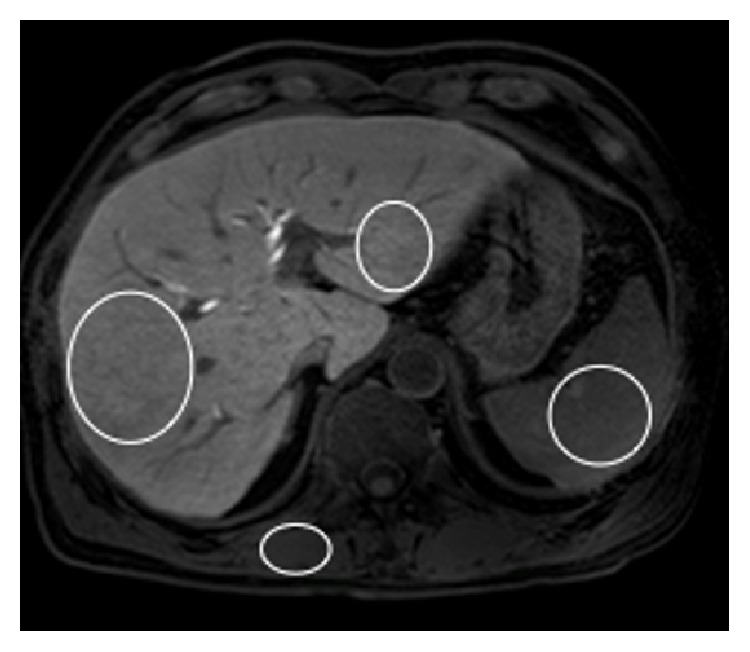
Hepatobiliary phase of Gd-EOB-DTPA-enhanced MRI. The signal intensities were measured by placing the largest possible regions of interest (ROIs) on the liver parenchyma, spleen, and erector spinae muscle, avoiding vessels, tumors, and artifacts. For the liver parenchyma, two round or oval ROIs were placed: one in the right lobe and the other in the left.

The signal intensity of axial eTHRIVE on Gd-EOB-DTPA-enhanced MRI was measured on the same DICOM viewer. First, two abdominal radiologists with six and 19 years of experience together selected three slices without significant artifacts. On the same slices they measured the signal intensities by placing the largest possible region of interest (ROI) on the liver parenchyma, spleen, and erector spinae muscle, avoiding vessels, tumors, and artifacts in a consensus manner (Figure 1). For the liver parenchyma, two round or oval ROIs were placed: one in the right lobe and the other in the left. The averages of the six signal intensities of the liver parenchyma and the three signal intensities of the spleen or the erector spinae muscle were calculated.

Based on these average values, the liver-to-spleen ratio (LSR) and the liver-to-muscle ratio (LMR) before and after the administration of Gd-EOB-DTPA were recorded for each patient. The same size and shape of ROI were placed at the same position for the images before and after the administration of Gd-EOB-DTPA. As indicators of liver function, the increase rates of the LSR (LMR) in the hepatobiliary phase compared with the precontrast image were calculated using the following equation: (LSR (LMR) on the hepatobiliary phase − LSR (LMR) on the precontrast image)/LSR (LMR) on the precontrast image [3, 4]. We named “the increase rate of LSR (LMR)” as “ΔLSR (ΔLMR).” We also set the parameter “ΔLSR (LMR) × LV” (unit; liter) for the analysis.

### 2.5. Pathologic Analysis

One pathologist with 4 years of experience who was unaware of the imaging data reviewed the hematoxylin-eosin-stained glass slides of each patient and referred to the official pathological report to determine the histological findings of the liver parenchyma. When the results were discordant, another experienced pathologist with 17 years of experience was consulted. The degree of liver fibrosis was classified into five groups according to the New Inuyama Classification: F0 (no fibrosis), F1 (fibrous portal expansion), F2 (bridging fibrosis), F3 (bridging fibrosis with architectural distortion), and F4 (liver cirrhosis) [9]. Similarly, the grade of necroinflammatory activity was scored as A0 (no necroinflammatory reaction), A1 (mild), A2 (moderate), and A3 (severe) [9].

### 2.6. Statistical Analysis

We used a linear regression analysis to examine the correlations between ΔLSR (ΔLMR) and ΔLSR (ΔLMR) × LV and the laboratory data corresponding to liver function (including Plt, Alb, T-bil, LDH, and ChE). The correlations of these four parameters with the Child-Pugh score, MELD score, the degree of liver fibrosis, and the grade of necroinflammatory activity were each examined using Spearman's rank correlation test. We also compared the correlation coefficients between ΔLSR and ΔLSR × LV and between ΔLMR and ΔLMR × LV. The statistical significance was evaluated using the following method: when the dependence of a variable (*y*, *z*) on a single independent variable (*x*) was observed, we calculated the correlation coefficient (*Rxy*, *Rxz*), and we tested the significance of the *Rxy*, *Rxz* coefficient by means of the modified *t*-test, the number of degrees of freedom being *f* = *n* − 3, using the following formula (*n* = sample  number):

(1) t-statistic=Ryz−Rxz·n−31+Ryz21−Rxy2−Rxz2−Ryz2+2RxyRyzRxz

(see [10]).

For all tests, a *p* value of <0.05 indicated a significant difference.

## 3. Results

The number of patients in each grade of fibrosis and necroinflammatory activity was as follows: F0 (*n* = 33), F1 (*n* = 11), F2 (*n* = 11), F3 (*n* = 12), and F4 (*n* = 37) and A0 (*n* = 30), A1 (*n* = 38), A2 (*n* = 32), and A3 (*n* = 4). The average LVs ± standard deviation (SD) in F0, F1, F2, F3, and F4 were 1.09 ± 0.24, 1.06 ± 0.26, 1.15 ± 0.17, 1.13 ± 0.26, and 1.06 ± 0.34, respectively. The average LVs ± SD in A0, A1, A2, and A3 were 1.08 ± 0.23, 1.04 ± 0.29, 1.13 ± 0.29, and 1.19 ± 0.30, respectively. The average values and SD of ΔLSR, ΔLSR × LV, ΔLMR, and ΔLMR × LV were 0.53 ± 0.30, 0.59 ± 0.37, 0.64 ± 0.29, and 0.70 ± 0.35, respectively. All four parameters (ΔLSR, ΔLSR × LV, ΔLMR, and ΔLMR × LV) were significantly correlated with all laboratory data, the grade of fibrosis, and necroinflammatory activity (*p* < 0.05 in each case).

Table 1 shows the correlation coefficients between ΔLSR or ΔLSR × LV and the laboratory data or pathologic factors. The correlation coefficient between ΔLSR × LV and ChE was significantly higher than that between ΔLSR and ChE (*p* < 0.05). The correlation coefficients between ΔLSR × LV and Plt, Alb, LDH, Child-Pugh score, or MELD score tended to be higher than those between ΔLSR and Plt, Alb, LDH, Child-Pugh score, or MELD score. However, the correlation coefficient between ΔLSR × LV and the degree of fibrosis or necroinflammatory activity was significantly lower than that between ΔLSR and the degree of fibrosis or necroinflammatory activity (*p* < 0.01). The correlation coefficient between ΔLSR × LV and T-bil tended to be lower than that between ΔLSR and T-bil.

**Figure 2:**
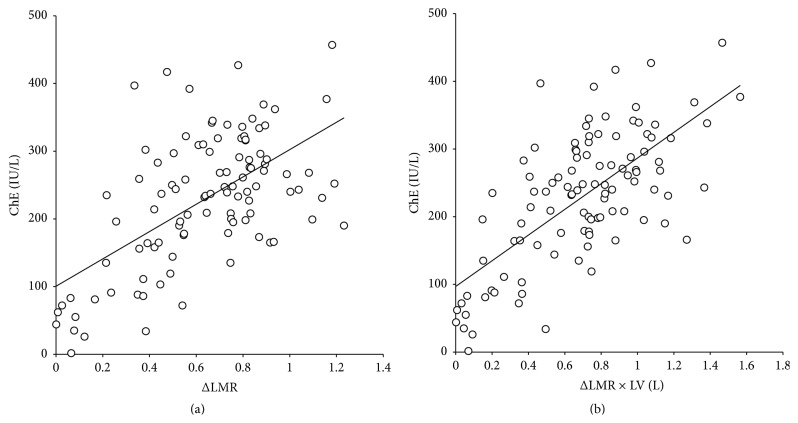
Scatterplot showing the relationship between (a) ΔLMR and ChE and (b) ΔLMR × LV and ChE (*n* = 103). (a) The regression analysis yielded the following standard formula (solid line): ChE = 201.9 ×  ΔLMR + 100.5 (correlation coefficient = 0.590; *p* < 0.01). (b) The regression analysis yielded the following standard formula (solid line): ChE = 189.7 ×  ΔLMR × LV + 97.0 (correlation coefficient = 0.681; *p* < 0.01). The correlation coefficient between ΔLMR × LV and ChE was significantly higher than that between ΔLMR and ChE.

Table 2 shows correlation coefficients between ΔLMR or ΔLMR × LV and the laboratory data or pathologic factors. The correlation coefficient between ΔLMR × LV and ChE was significantly higher than that between ΔLMR and ChE (*p* < 0.01) (Figure 2). The correlation coefficients between ΔLMR × LV and Plt, Alb, LDH, Child-Pugh score, or MELD score tended to be higher than those between ΔLMR and Plt, Alb, LDH, Child-Pugh score, or MELD score. However, the correlation coefficient between ΔLMR × LV and the degree of fibrosis or necroinflammatory activity was significantly lower than that between ΔLMR and the degree of fibrosis (*p* < 0.05) or necroinflammatory activity (*p* < 0.01). The correlation coefficient between ΔLMR × LV and T-bil tended to be lower than that between ΔLMR and T-bil.

## 4. Discussion

In our study using 3T-MRI, significant correlations between the uptake of Gd-EOB-DTPA and liver function, fibrosis, and necroinflammatory activity were obtained, as reported previously [4–7]. In light of this result, we feel that our radiological assessment is valid for evaluating liver function, fibrosis, and necroinflammatory activity. In addition, the correlation coefficient between ΔLSR (LMR) × LV and ChE was significantly higher than that between ΔLSR (LMR) and ChE. The correlation coefficients between ΔLSR (LMR) × LV and Plt, Alb, LDH, Child-Pugh score, or MELD score tended to be higher than those between ΔLSR (LMR) and Plt, Alb, LDH, Child-Pugh score, or MELD score, suggesting that we should consider “liver volume” in addition to the uptake of Gd-EOB-DTPA for setting the MR parameters. Recently, some articles have reported that the relationship between the uptake of Gd-EOB-DTPA and indocyanine* *green* *test can be improved by considering liver volume [11–13] and supports our result or hypothesis.

In general, liver function data are evaluated with a blood test, which includes a “whole liver” element. Therefore, the consideration of liver volume in the MR parameter could enable the correlation with liver function to be more intensive. We found in the present study that the correlation coefficient between ΔLSR (LMR) × LV and T-bil tended to be lower than that between ΔLSR (LMR) and T-bil, although the difference was only slight. T-bil includes both unconjugated and conjugated bilirubin, and the T-bil value can be affected by a number of factors including prehepatic or posthepatic disorders, hemolysis, and constitutional predisposition. Therefore, considering “liver volume” in the MR parameter might not be effective for the correlation with T-bil.

We also found that the correlation coefficients between ΔLSR (LMR) × LV and the degree of fibrosis or necroinflammatory activity were significantly lower than those between ΔLSR (LMR) and the degree of fibrosis or necroinflammatory activity. That is, the consideration of liver volume in addition to the uptake of Gd-EOB-DTPA for setting the MR parameters was not useful. Although this result was beyond the scope of our hypothesis, we propose two plausible reasons why this result was obtained. One is that fibrosis and necroinflammatory activity represent the local state of the liver parenchyma. Therefore, the consideration of “liver volume” might worsen the correlation with liver pathology. Another possible reason is that the LV does not always decrease gradually as the degree of fibrosis progresses. A report on LV change in patients with hepatic fibrosis is available [14]. The LV tends to increase with the severity of fibrosis since the number of hepatic cells accounts for 70%–80% of the liver parenchyma and then decrease. The presumed reason for the hepatic volume increase would be the ballooning of hepatocytes along with the increased fibrotic component.

We obtained a similar result; that is, LV tends to increase with the severity of fibrosis from F0 to F2 but decrease at F3 to F4, which would affect the rank correlation between ΔLSR (LMR) × LV and the degree of fibrosis. It was reported that the LV tends to increase with the aggravation of inflammatory activity (the increase of necroinflammatory activity) [14]. In our study we obtained a similar result; that is, the LV tends to increase as the degree of necroinflammatory activity advances from A1 to A3. Therefore, the LV consideration would have the opposite effect on the correlation with the degree of necroinflammatory activity. We thus suggest that “liver volume” should not be considered among the MR parameters when evaluating liver pathology using Gd-EOB-DTPA-enhanced MRI.

Our study had several limitations. First, the trial was a study with a limited patient population, and the number of cases with each degree of fibrosis and necroinflammatory activity was not uniform. Second, we used two organs, the spleen and erector spinae muscle, as signal intensity references of the liver parenchyma. As there may be persistence of contrast enhancement in the spleen and muscle, these organs might be limitations for analyses of LSR and LMR as well as motion artifacts and partial volume effects. Although a T1 map might be preferable for the quantitative analysis of the uptake of Gd-EOB-DTPA, it was difficult to generate such a map with our scanner. Third, we could not evaluate indocyanine* *green* *test results as a laboratory datum corresponding to liver function. Although 80 patients underwent this test preoperatively, the Child-Pugh classification for all of them was Grade A. That is, patients with moderate or severe liver dysfunction were not included. We judged that we should not juxtapose the comparison with ICG test to those with other liver function parameters in our study, because of the difference in patient population. Finally, tumor volumes of small lesions in the liver were not excluded from measured LV for technical difficulty, which may have led to minor overestimation of LV in some patients.

## 5. Conclusion

We have demonstrated that the inclusion of liver volume may improve Gd-EOB-DTPA-based predictions of liver function, but not in predictions of liver pathology.

## Figures and Tables

**Table 1:** Correlation coefficients between ΔLSR or ΔLSR × LV and the laboratory or pathologic data.

Parameter	ΔLSR	ΔLSR × LV	*p* value
Plt	0.498	0.522	0.49
Alb	0.624	0.646	0.49
T-bil	0.364	0.330	0.40
LDH	0.238	0.244	0.88
ChE	0.577	0.649	<0.05
Child-Pugh score	−0.592	−0.641	0.12
MELD score	−0.471	−0.478	0.85

Fibrosis	−0.492	−0.383	<0.01
Necroinflammation	−0.451	−0.341	<0.01

The data are correlation coefficients. LSR: the liver-to-spleen ratio; ΔLSR: the increase rate of LSR on the hepatobiliary phase compared with the precontrast image. Plt: platelet count; Alb: albumin; T-bil: total bilirubin; LDH: lactate dehydrogenase; ChE: cholinesterase. ΔLSR and ΔLSR × LV were calculated as described in Section 2.

**Table 2:** Correlation coefficients between ΔLMR or ΔLMR × LV and the laboratory or pathologic data.

Parameter	ΔLMR	ΔLMR × LV* *	*p* value
Plt	0.405	0.457	0.22
Alb	0.668	0.701	0.22
T-bil	0.400	0.382	0.69
LDH	0.211	0.249	0.41
ChE	0.590	0.681	<0.01
Child-Pugh score	−0.599	−0.655	0.12
MELD score	−0.433	−0.477	0.29

Fibrosis	−0.493	−0.395	<0.05
Necroinflammation	−0.462	−0.324	<0.01

The data are correlation coefficients. LMR: the liver-to-erector spinae muscle; ΔLMR: the increase rate of LMR on the hepatobiliary phase compared with the precontrast image. ΔLMR and ΔLMR × LV were calculated as described in Section 2.
